# Real-world drug survival of patients with hidradenitis suppurativa treated with adalimumab

**DOI:** 10.1016/j.jdin.2023.03.004

**Published:** 2023-03-29

**Authors:** Ryan C. Saal, Sarah Alnaif, Joshua F. Edwards, Jennifer A. Wintringham, Robert J. Smith

**Affiliations:** Department of Dermatology, Eastern Virginia Medical School, Norfolk, Virginia

**Keywords:** adalimumab, biologics, biologic longevity, hidradenitis suppurativa, hidradenitis suppurativa management, real-world effectiveness

*To the Editor:* Adalimumab is the only Food and Drug Administration–approved biologic medication to treat hidradenitis suppurativa (HS) as of March 31st, 2023. Although the results of many studies demonstrate the efficacy of adalimumab for the treatment of HS, the degree of improvement is significantly less than patients with other diseases treated with adalimumab (eg, psoriasis and rheumatoid arthritis).^1^, ^2^, ^3^ The real-world drug survival of patients who require transition from adalimumab to another biologic therapy or cease all biologic therapy has not been well described.

We identified 412 patients with HS from June 1, 2014 to January 1, 2021 treated at a single, tertiary care, academic dermatologic practice. Among patients with a minimum of 3 visits to our practice (n = 134), 41 (31%) were treated with a biologic agent. Of these patients, 29 (71%) were biologic naïve before establishment of care with the practice. Table I shows patient demographics and characteristics of this cohort. Average age ± SD was 39.17 ± 12.37 years. Demographically, 25 (86%) patients were women, 26 (90%) were Black or African American, and 15 (52%) had private insurance. Overall, 11 (38%) patients noted a HS diagnosis of >10 years and 14 (56%) were a current or former smoker. Average body mass index score was 35.70 ± 8.83 (Table I). The demographic characteristics of the biologic-naïve patients were not significantly different than the greater cohort of 412 patients with HS.

**Table I:** Patient demographics and characteristics (n = 29)

Variable	Count (%)
Age (mean ± SD)	39.17 ± 12.37
Sex	
Male	4 (14)
Female	25 (86)
Race	
Black or African American	26 (90)
Hispanic	1 (3)
Multiracial	1 (3)
White	1 (3)
Insurance status	
Government-Assisted Insurance (Medicare, Medicaid, TriCare)	13 (45)
Private Insurance	15 (52)
Unknown	1 (3)
Smoking history	
Current smoker	13 (52)
Never smoker	11 (44)
Previous smoker	1 (4)
No. of years with a history of HS diagnosis, ys	
<1	2 (7)
1-5	9 (31)
5-10	3 (10)
>10	11 (38)
Unspecified amount of years	4 (14)
Hurley stage	
1	1 (3)
2	4 (14)
3	8 (28)
Not Documented	16 (55)
BMI	35.70 ± 8.83
Average length of follow-up during study period	2.75 ± 2.14
Total length of time on adalimumab therapy (years)	1.34 ± 0.99
Biologics started by EVMS Dermatology	
Adalimumab	28 (97)
Infliximab	1 (3)
If a patient was prescribed their first biologic medication for HS at EVMS, what was the visit no. (1 = initial HS visit with EVMS Dermatology)?	
1	17 (59)
2	5 (17)
3	3 (10)
4	1 (3)
6	2 (7)
15	1 (3)

*BMI*, Body mass index; *EVMS*, Eastern Virginia Medical School; *HS*, hidradenitis suppurativa.

Among the subset of patients for whom a biologic was initiated for their HS at this practice (n = 29), 17 (59%) were prescribed a biologic at their first visit. Of these patients with HS initiated on a biologic agent, 28 (97%) were initiated on adalimumab. Mean duration of adalimumab therapy by the end of the study period was 1.34 ± 0.99 years. In addition, 10 (36%) patients ultimately discontinued adalimumab, whereas 18 (64%) continued adalimumab therapy. Of the 10 (36%) patients that discontinued biologic therapy, the mean duration of adalimumab therapy was 1.17 ± 0.90 years. The primary rationale for adalimumab cessation was cited as lack of clinical response (6 [60%]), adverse side effect (3 [30%]), and cost of medication/insurance coverage (1 [10%]). Moreover, 5 (50%) patients were then transitioned to another biologic, whereas 5 (50%) opted to completely discontinue biologic therapy (Fig 1). The variables of age, sex, race, insurance type, smoking history, and Hurley stage were not predictive of adalimumab cessation or continuation (Fig 1). Limitations of our study include a small sample size.

**Fig 1:**
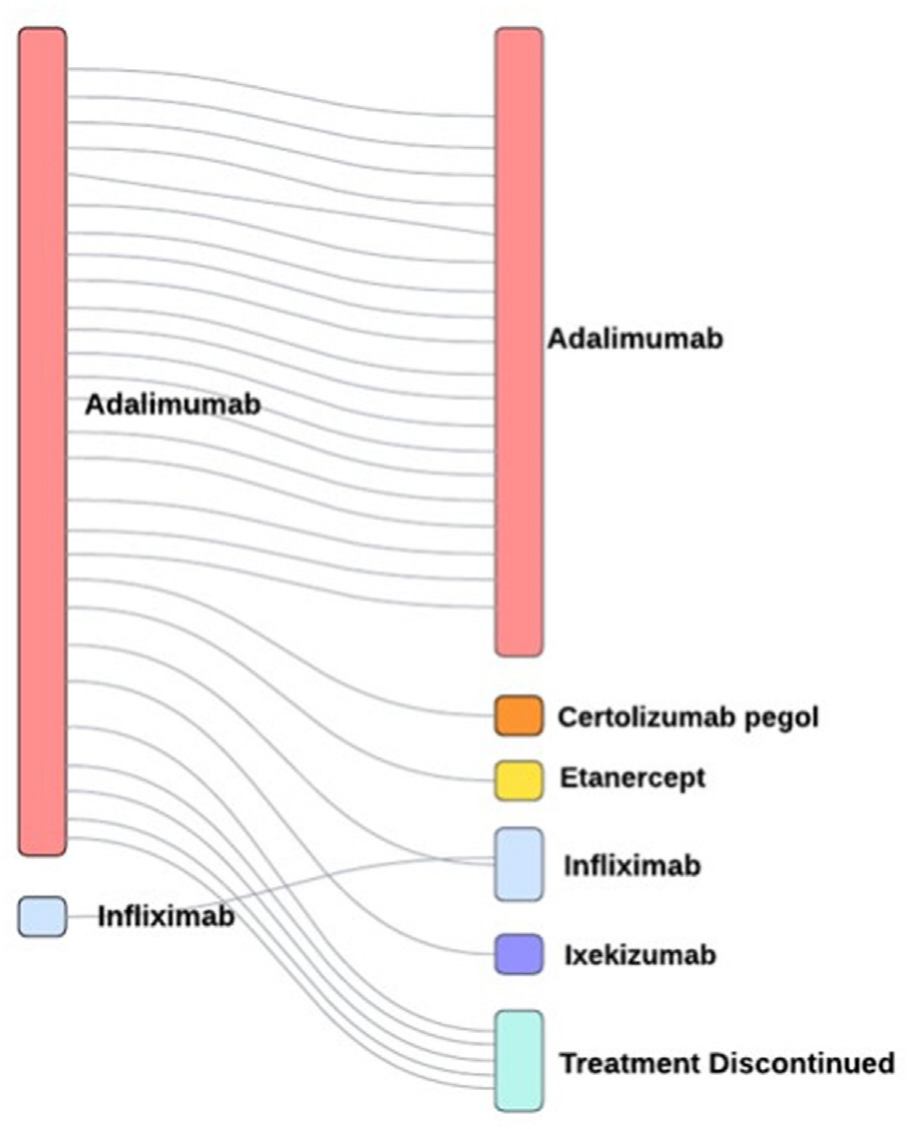
Visual representation of the course of hidradenitis suppurativa treatment for biologic-naïve patients initiated on a biologic.

Biologic treatments are an increasingly important tool in the management of patients with moderate-to-severe HS. Among the patients initiated biologic treatment, a moderate percentage of patients ceased therapy or necessitated transition to another agent. Our retrospective study highlights the variability of real-world responsiveness to adalimumab among patients with HS. A recent nationwide study has found that HS drug survival is decreased compared with psoriasis, inflammatory bowel disease, or rheumatoid arthritis.^4^ In addition, in the treatment of HS, adalimumab has decreased drug survival compared with infliximab, ustekinumab, and etanercept.^4^

## Conflicts of interest

None disclosed.
